# Ferratin

**Published:** 1895-01-12

**Authors:** 


### NEW DRUGS AND PREPARATIONS.

[We shall be glad to receive, at our Office, 428, Strand, London, W.C., from the manufacturers, specimens of all new preparations and drugs which may be brought out from time to time.]

### FERRATIN.

Further observations with ferratin serve to confirm its value as a hæmatinic, although the results, in so far as formation of hæmoglobin and red corpuscles is concerned, do not appear to be superior to those obtained with good inorganic preparations of iron. It is an organic iron compound, containing about 7 per cent. of iron, and is perfectly unirritating, being therefore specially adapted for those cases of anæmia in which the stomach is very irritable. It is said to be particularly valuable in cases of dyspepsia where there is a slight tendency to anæmia, without the latter ever becoming very pronounced, as it supplies iron in a small compass, and in the same kind of combination in which it is present in our food. Mr. John Harold[1] gives particulars of three cases of typical chlorosis treated by it. The patients received doses of 8 to 30 grains three times daily, and all of them showed rapid and marked improvement from the beginning, but it took from six to eight weeks to restore the blood condition to comparative health, a result certainly not superior to what can be got from treatment with pharmacopœial preparations of iron. Germain Sée[2] states that it acts as a slight astringent to the alimentary canal without producing irritation, so that it improves appetite and digestion. It is but slowly absorbed, and the dosage should be so regulated that there is always a surplus in the bowel, on which the body may draw. Ferratin is therefore a food, and may be given as such in all conditions in which an excess of alimentary iron seems indicated. He gives from about 1 to 22 grains twice or thrice daily, according to the requirements of the patient.

[1] Practitioner, Aug., 1894. [2] Presse Méd., Aug. 25th, 1894.

